# To monitor the COVID-19 pandemic we need better quality primary care data

**DOI:** 10.3399/bjgpopen20X101070

**Published:** 2020-04-16

**Authors:** Simon de Lusignan, John Williams

**Affiliations:** 1 Nuffield Department of Primary Care Health Sciences, University of Oxford, Oxford, UK

**Keywords:** Practice organisation, primary healthcare, general practice, coronavirus

**​UK primary care coding of covid-19 is a mess: we need to stop the use of bad codes, and migrate from the use of ugly to good codes, but will only be able to do so when they are finally released** **.**

Key data computerised medical record (CMR) systems are recorded using ’codes’, to standardise recording and so attendances about a medical problem can be linked.^1^ At the start of the COVID-19 pandemic there was neither international agreement about nomenclature nor codes available in primary care CMRs with which to record exposure, testing, or infection.

We have now been through three iterations of clinical codes in the UK since the end of January. Five temporary codes were added to all the primary care CMR systems using the *‘2019 nCoV (Wuhan)’* label in January 2020. Subsequently NHS Digital, the NHS coding organisation, released a more extensive set of SNOMED CT concepts named *‘2019 nCoV (novel coronavirus*)’ because the use of ‘Wuhan’ had been deprecated; these codes were in turn replaced by *‘SARS –CoV-2 (severe acute respiratory syndrome coronavirus 2)’* ^2^

The situation has been further complicated by the fact that this last release is only now starting to become available in CMRs (Table 1), and because some clinicians have gone back to using old non-specific coronavirus codes (such as *‘* *Suspected Coronavirus infection: 1JX* *’*, and *‘* *Coronavirus infection: A795* *’*).

**Table 1.** Clinical concepts that should be coded, temporary and definitive codes

**Clinical concepts that should be coded in CMR**	**Temporary codes** ***Go on using until replaced by SARS-Cov-2***	**Final SNOMED CT description *Roll-out taking place during April 2020***
Exposure to COVID-19	Exposure to 2019 nCoV (Wuhan) infection *or*	Exposure to SARS-CoV-2 infection
	Exposure to 2019 nCoV (novel coronavirus) infection	
Suspected COVID-19 infection	Suspected 2019 nCoV (Wuhan) infection *or*	Suspected COVID-19
	Suspected 2019 nCoV (novel coronavirus) infection	
Test for COVID-19 offered or taken	*No specific codes*	Swab for SARS-CoV-2 (severe acute respiratory syndrome coronavirus 2) taken by healthcare professional
		Self-taken swab for SARS-CoV-2 (severe acute respiratory syndrome coronavirus 2) offered
		Self-taken swab for SARS-CoV-2 (severe acute respiratory syndrome coronavirus 2) completed
	Tested for 2019 nCoV (Wuhan) infection *or*	
	Tested for 2019 nCoV (novel coronavirus) infection	
COVID-19 definite case	Confirmed 2019 nCoV (Wuhan) infection *or*	COVID-19
	Confirmed 2019 nCoV (novel coronavirus) infection	
COVID-19 excluded	Excluded 2019 nCoV (Wuhan) infection *or*	COVID-19 excluded
	Excluded 2019 nCoV (novel coronavirus) infection	
**Laboratory test codes**		
COVID-19 confirmed by lab test		COVID-19 confirmed by laboratory test
COVID-19 excluded by lab test		COVID-19 excluded by laboratory test
COVID-19 virus detected	2019-nCoV (novel coronavirus) detected	SARS-CoV-2 (severe acute respiratory syndrome coronavirus 2) detected
COVID-19 virus not detected	2019-nCoV (novel coronavirus) not detected	SARS-CoV-2 (severe acute respiratory syndrome coronavirus 2) not detected

CMR = computerised medical record.

This creates challenges for the surveillance system and others monitoring the pandemic.^3^ We have previously classified the incorrect use of codes as miscoding, misclassification, or misdiagnosis.^4^ In the cases of COVID-19, we are seeing^1^ both **Miscoding** (that is, continued use of the temporary codes, which should stop once the new ones are available);^2^ and **Misclassification** (use of non-specific coronavirus codes), which should stop. Table 1 sets out the clinical concept we currently need to consistently record in primary care, the temporary codes available to do this, and the final codes we should all eventually use. Prompt cards to help clinicians and coders are available at: https://clininf.eu/index.php/cov-19/

All UK primary care clinicians and coders are recommended to continue to use the temporary codes until the new ones are available, then switch. Accurate data is a key to understanding and monitoring the course of this pandemic.

**Appendix: Examples of codes not to use**

- Exposure to coronavirus infection
- Suspected coronavirus infection
- Coronavirus infection
- Disease due to Coronaviridae
- Coronavirus contact
